# Luseogliflozin Suppresses Late-Night Basal Glycemic Variability in Type 2 Diabetes: A Retrospective Observational Study

**DOI:** 10.7759/cureus.95930

**Published:** 2025-11-02

**Authors:** Keishi Yamauchi

**Affiliations:** 1 Department of Diabetes and Endocrinology, International University of Health and Welfare Shioya Hospital, Yaita, JPN

**Keywords:** coefficient of variation, diabetes, glycemic variability, late-night, sglt2 inhibitor

## Abstract

Introduction: Glucose fluctuations have been implicated in the development of diabetic macroangiopathy. Sodium-glucose cotransporter 2 (SGLT2) inhibitors are known not only for their glucose-lowering effects but also for their protective effects on the kidneys and heart. Basal glycemic variability (GV) during late-night periods, when external factors such as meals and physical activity are minimized, may represent a clinically important marker of intrinsic glucose regulation. However, little is known about the impact of SGLT2 inhibitors on basal GV. This study investigated the acute (within 24 hours) effect of the first administration of luseogliflozin, an SGLT2 inhibitor, on nocturnal basal GV in patients with type 2 diabetes mellitus.

Patients and methods: Ten patients with type 2 diabetes who met the criteria were retrospectively selected. Luseogliflozin (2.5 mg/day) was initiated after baseline assessment. Continuous glucose monitoring (CGM; FreeStyle Libre Pro, Abbott Diabetes Care Inc., Alameda, California) was performed, and the coefficient of variation (CV) of glucose was calculated as an index of glycemic variability (GV). Nocturnal basal GV was evaluated between 11:00 P.M. and 3:00 A.M. on the night before and the night after the first administration. This study was retrospective because CGM data before and after luseogliflozin initiation were obtained from existing hospital records, without prior intent to evaluate this specific effect.

Results: CGM revealed heterogeneous nocturnal glucose patterns with small but clinically relevant fluctuations. Luseogliflozin significantly reduced nocturnal glucose CV from 11.9% ± 3.8% at baseline to 7.2% ± 4.1% after treatment (p = 0.0048). These findings indicate that even after the first dose, luseogliflozin may acutely stabilize basal glucose fluctuations during late-night periods.

Conclusion: This is the first study to demonstrate that an SGLT2 inhibitor can suppress nocturnal basal GV immediately after initiation. Given that GV has been associated with sympathetic activation and vascular injury, these results suggest that reducing basal GV with SGLT2 inhibitors may contribute to vascular protection. Further studies with larger sample sizes and longer follow-up are warranted to confirm these observations.

## Introduction

Type 2 diabetes mellitus (T2D) is a major global health problem characterized by chronic hyperglycemia and progressive vascular complications. Beyond mean glucose levels, glycemic variability (GV), the degree of glucose fluctuations over time, has emerged as an important clinical marker. Previous studies have shown that GV is independently associated with hypoglycemia risk, cardiovascular disease, microvascular complications, and overall mortality in patients with diabetes [1-4]. This suggests that GV may contribute to diabetic complications through mechanisms such as oxidative stress, endothelial dysfunction, and sympathetic overactivity [5,6]. Furthermore, nocturnal basal GV is clinically important because it reflects endogenous glucose regulation in a relatively stable state, unaffected by food intake, physical activity, or acute stress. Excessive fluctuations during late-night periods may exacerbate glycemic instability, provoke sympathetic activation, and accelerate vascular injury [5,6].

Sodium-glucose cotransporter 2 (SGLT2) inhibitors lower blood glucose by enhancing urinary glucose excretion, and they are well established for their cardiovascular and renal protective benefits [7]. In addition, clinical studies indicate that SGLT2 inhibitors can improve certain aspects of GV, including postprandial glucose excursions [8,9]. However, while several trials and meta-analyses have addressed their impact on overall 24-h GV, evidence regarding their effect on basal (fasting or nocturnal) GV remains scarce [10].

Therefore, the objective of this study was to evaluate the acute (within 24 hours) effect of luseogliflozin, assessed immediately after the first administration, on nocturnal basal GV in patients with T2D using continuous glucose monitoring (CGM). By focusing on this specific timeframe, the study aimed to clarify whether SGLT2 inhibition can stabilize basal glucose fluctuations and contribute to vascular protection.

Part of this work was previously presented as a poster at the 81st American Diabetes Association Annual Scientific Sessions from June 25 to 27, 2021.

## Materials and methods

Patients

A total of 63 patients with T2DM were admitted for diabetes education. This single-center, retrospective observational study was conducted at International University of Health and Welfare Shioya Hospital (Tochigi, Japan), between September 2019 and March 2020. The study was approved by the Ethics Committee of the International University of Health and Welfare Shioya Hospital (approval no. 13-B-393).

Consecutive patients admitted during the study period were screened. Inclusion criteria were 1) age ≥18 years and 2) confirmed diagnosis of T2D according to the Japan Diabetes Society criteria. Exclusion criteria were 1) HbA1c >9%, 2) use of any antidiabetic drug except metformin, 3) history of liver disease, 4) history of pancreatic disease, 5) renal dysfunction (eGFR ≤ 45 mL/minute/1.73 m²), and 6) diagnosis or treatment of insomnia. Of the 63 patients, 53 were excluded (31 using other antidiabetic agents except metformin, six with liver disease, four with pancreatic disease, five with renal dysfunction, and seven with insomnia). Thus, 10 patients were included in the final analysis. All patients admitted to our hospital for the diabetes education program were fitted with CGM devices for inpatient glucose management. Among them, luseogliflozin was administered to those without contraindications or conditions requiring cautious use, as it was the only SGLT2 inhibitor available in our hospital at that time and had demonstrated short-term efficacy.

Luseogliflozin (2.5 mg daily, administered in the morning 30 minutes after breakfast) was initiated on day 3 after admission. Routine blood sampling, including serum C-peptide (CPR), was performed at 6:00 A.M. in the fasting state. Luseogliflozin (2.5 mg daily, administered in the morning 30 minutes after breakfast) was initiated on day 3 after admission, after two days of baseline CGM data collection. Routine blood sampling, including serum CPR, was performed at 6:00 A.M. in the fasting state, which is the standard morning sampling time at our hospital. Luseogliflozin was well tolerated. Missing data were minimal, except for one patient in whom fasting CPR was not obtained.

Study design

The coefficient of variation (CV), which normalizes variability relative to mean glucose, was used as the primary index of GV. CV was one of the indices of GV [11]. Lazar et al. [11] list several indices of blood glucose fluctuation and describe the advantages and disadvantages of each. CV is obtained by dividing the standard deviation (SD) by the average of the glycemic values, thus serving as a GV index adjusted for the mean glucose level [12].

$\mathrm{CV}(\%) = \frac{\mathrm{SD}}{\text{mean glucose}} \times 100$

Since SGLT2 inhibitors reduce overall blood glucose levels, using SD alone may underestimate changes in GV. Because the SD tends to increase proportionally with the mean, CV is considered a more appropriate measure of variability in this context. Therefore, in this study, CV was selected as the primary index because it reflects relative variability and is not affected by the overall blood glucose level.

All patients were hospitalized and received a standardized diabetic diet, ensuring adherence and minimizing dietary and lifestyle influence. Data were collected from the third to fourth day of hospitalization, when the effect of the preadmission was considered minimal.

Nocturnal basal GV was assessed between 11:00 P.M. and 3:00 A.M. on the night before and the night after the first administration of luseogliflozin. This time window was chosen because it is minimally affected by meals, physical activity, or stress, allowing evaluation of basal glucose fluctuations under stable physiological conditions. In addition, blood samples were routinely taken at 11:00 P.M. and 3:00 A.M. during hospitalization on other days, which is why this period was chosen for evaluating stable nocturnal glucose changes.

Although the study was retrospective, this time window enabled objective comparison of CGM data recorded before and after the initial dose of luseogliflozin. This study was retrospective because the effect of luseogliflozin on nocturnal GV was not anticipated in advance, and a prospective protocol with ethical approval had not been established. At the time, luseogliflozin was the only SGLT2 inhibitor available in our hospital, and it was therefore selected for analysis. Since then, multiple SGLT2 inhibitors have been introduced into our practice, making it difficult to reproduce the same conditions in a retrospective manner. The final sample size was determined by the number of eligible patients during the study period, and a post hoc power analysis was performed for the primary outcome (nocturnal glucose CV). This approach is consistent with previous studies demonstrating that SGLT2 inhibitors can exert measurable metabolic effects within 24-48 hours after administration [13,14].

Statistical analysis

Continuous variables are presented as the mean ± SD or as the median and interquartile range, as appropriate. Differences between paired groups were assessed using the paired Student’s t-test. The Wilcoxon matched-pairs test or the Mann-Whitney U test was used for nonparametric data. A two-sided p value of <0.05 was considered statistically significant. Statistical analyses were conducted using Easy R version 4.02 (Saitama Medical Center, Jichi Medical University, Saitama, Japan).

## Results

The participants (male:female ratio, 6:4) had a mean age of 54.3 ± 12.8 years, a mean body mass index (BMI) of 27.3 ± 5.7 kg/m², and a mean diabetes duration of 3.6 ± 1.6 years. At baseline, HbA1c was 7.6% ± 1.5%, indicating moderately controlled diabetes. After luseogliflozin administration, fasting plasma glucose showed a modest reduction (from 8.6 ± 1.4 to 7.5 ± 1.0 mmol/L), but this difference did not reach statistical significance (p = 0.058). In contrast, average nocturnal glucose between 11 P.M. and 3 A.M. significantly decreased (from 8.53 ± 1.01 to 7.34 ± 0.53 mmol/L, p = 0.023). These results are shown in Table 1.

**Table 1:** Baseline characteristics and changes before and after luseogliflozin treatment The baseline characteristics of the patients, as well as changes in fasting blood glucose and late-night CGM values before and after luseogliflozin administration, are shown along with p values. Values are presented as mean ± SD unless otherwise indicated. p values were calculated using a paired t-test. A p value of <0.05 was considered statistically significant ^*^CPR data were missing for one patient eGFR: estimated glomerular filtration rate; HbA1c: glycated hemoglobin; CGM: continuous glucose monitoring; CV: coefficient of variation; SD: standard deviation; CPR: C-peptide

Parameter	Before	After	p value
Male:female ratio	6:4	-	-
Mean age (years)	54.3 ± 12.8	-	-
Body mass index (kg/m²)	27.3 ± 5.7	-	-
Diabetes duration (years)	3.6 ± 1.6	-	-
Metformin use (mg/day)	4 (40%); 937.5 ± 427.0	-	-
eGFR (mL/minute/1.73 m^2^)	74.7 ± 16.9	-	-
HbA1c (%)	7.6 ± 1.5	-	-
Fasting plasma glucose (mmol/L)	8.6 ± 1.4	7.5 ± 1.0	0.058
Fasting CPR (ng/mL)^*^	4.26 ± 1.92	-	-
Average glucose level by CGM (11 P.M. to 3 A.M.) (mmol/L)	8.53 ± 1.01	7.34 ± 0.53	0.023
CV by CGM (11 P.M. to 3 A.M.)	11.9 ± 3.8	7.2 ± 4.1	0.0048

**Figure 1:**
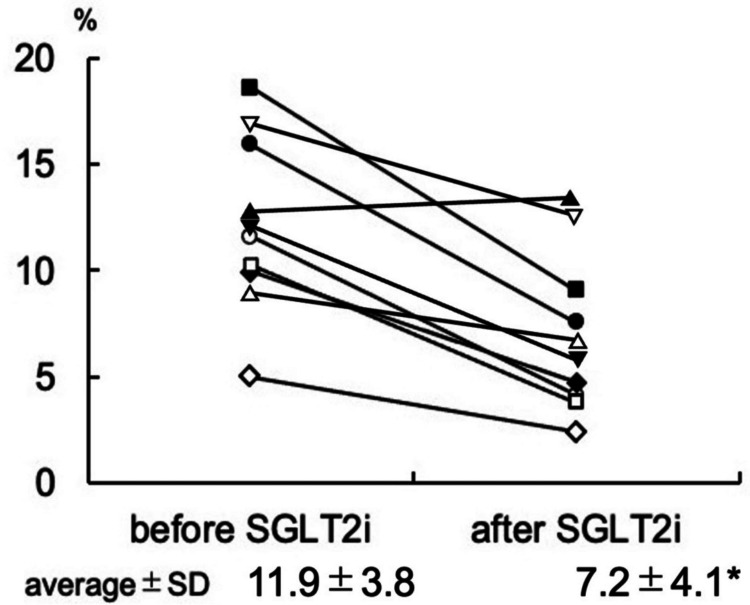
Effect of luseogliflozin on nocturnal glucose variability The CV of glucose between 11:00 P.M. and 3:00 A.M. was significantly reduced following treatment. CV, calculated as the ratio of SD to mean glucose, was used as an index of glycemic variability; a decrease in CV indicates reduced variability. Values are presented as mean ± SD. The p value was derived from a paired t-test ^*^p = 0.0048 vs. baseline before luseogliflozin administration. A p value of <0.05 was considered statistically significant SD: standard deviation; SGLT2i: sodium-glucose cotransporter 2 inhibitor; CV: coefficient of variation; SD: standard deviation

CGM revealed small but distinct fluctuations in glucose levels, even during late-night periods. Luseogliflozin significantly reduced glucose CV (from 11.9% ± 3.8% to 7.2% ± 4.1%, p = 0.0048), indicating suppression of fine nocturnal fluctuations. These findings are illustrated in Figure 1.

Based on the observed reduction in glucose CV (from 11.9 ± 3.8 to 7.2 ± 4.1), the effect size (mean paired difference / SD of paired differences) was estimated at 1.15. With a two-sided α of 0.05, the achieved power exceeded 80% using a paired Student’s t-test. These results indicate that, despite the small sample size, the study had sufficient power to detect the observed effect.

**Figure 2:**
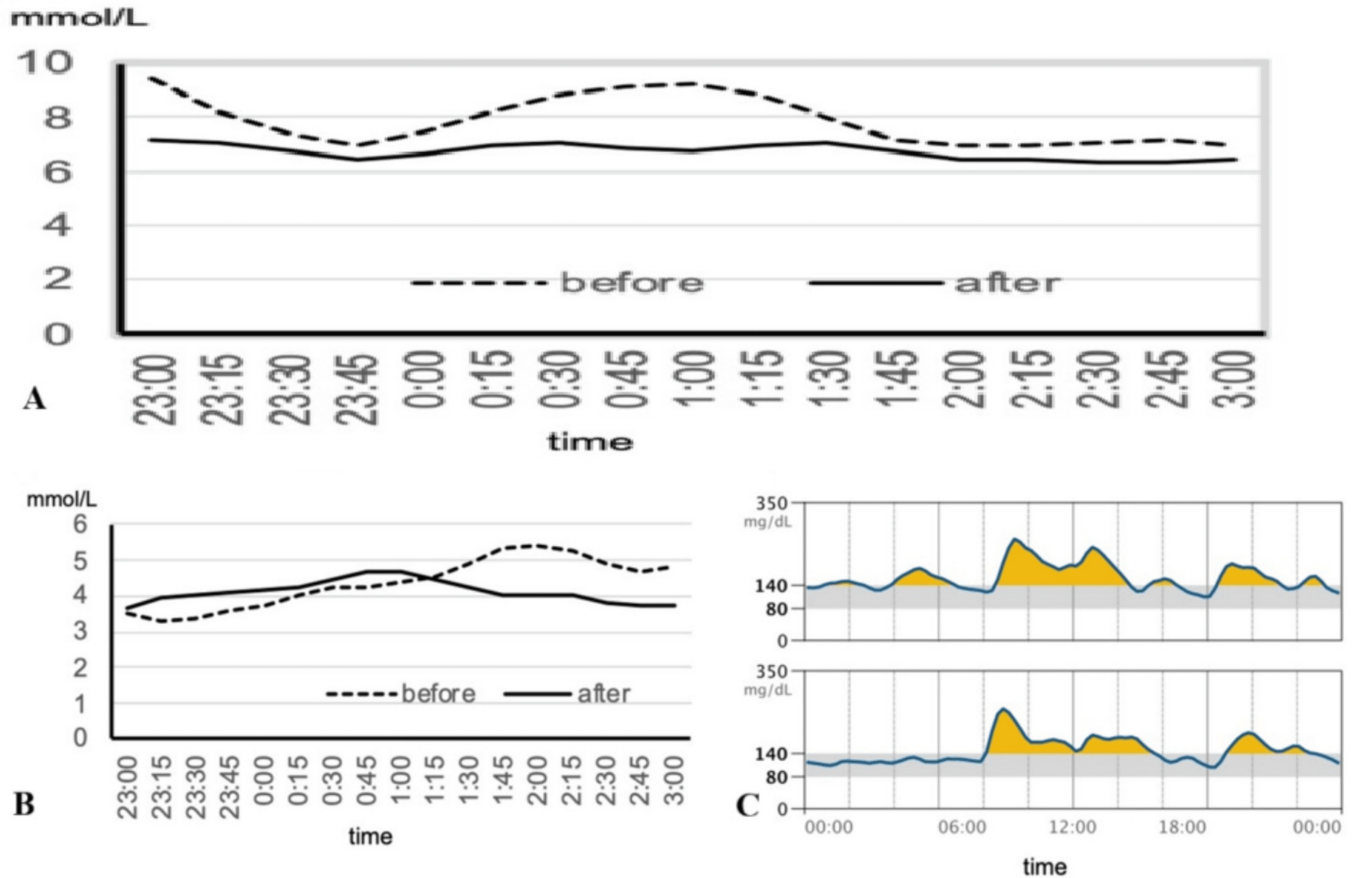
Representative CGM profiles before and after luseogliflozin treatment (A,B) Late-night CGM glucose profiles in two representative subjects, illustrating suppression of nocturnal fluctuations and attenuation of a progressive nocturnal rise. Glucose levels are expressed in mmol/L. (C) 24-hour CGM data from another subject, demonstrating preserved daytime variability with marked suppression of nocturnal fluctuations. Glucose levels are expressed in mg/dL CGM: continuous glucose monitoring

Late-night CGM glucose profiles in representative subjects demonstrated characteristic changes after luseogliflozin treatment. In one case, nocturnal glucose fluctuations were clearly suppressed (Figure 2A), while in another, the progressive rise in glucose levels during the night was blunted (Figure 2B). Furthermore, 24-hour CGM data from a different subject showed that daytime glucose fluctuations remained largely unchanged, whereas nocturnal fluctuations were markedly suppressed (Figure 2C).

Overall, basal GV was promptly reduced following luseogliflozin administration. Nighttime mean glucose levels decreased, but the reduction in SD was even greater, resulting in a substantial decrease in CV. This finding suggests a specific suppression of basal fluctuations. In contrast, 24-hour CV did not change significantly, indicating that daytime variability is more strongly affected by meals and stress. As previously reported [9], SGLT2 inhibitors lower both pre- and postprandial glucose levels but exert only a limited effect on overall GV. This inhibitory effect on basal variability was not observed with sulfonylureas or dipeptidyl peptidase 4 inhibitors (data not shown). Taken together, these results indicate that luseogliflozin effectively suppresses basal glycemic fluctuations.

## Discussion

SGLT2 inhibitors lower mean glucose levels, but their ability to improve GV has been considered limited [9]. The present findings, showing that luseogliflozin suppresses nocturnal GV, provide novel insight into the pleiotropic effects of SGLT2 inhibitors. A recent meta-analysis demonstrated that SGLT2 inhibitors modestly reduce short-term GV, although the effect size varied depending on the specific drug and patient background [9]. Thus, our study contributes additional evidence that SGLT2 inhibitors may influence basal GV beyond their average glucose-lowering effect.

Diabetes is a leading cause of microvascular complications such as nephropathy, retinopathy, and peripheral neuropathy [15,16]. It is also associated with accelerated atherosclerosis and cardiovascular disease [17], which accounts for about 50% of deaths in patients with T2D [18,19]. Long-term GV has been shown to predict cardiovascular events and mortality independently of mean HbA1c [6,7]. Therefore, stabilizing GV, particularly during basal periods such as midnight, may represent a clinically relevant therapeutic target.

The biological mechanisms linking GV to vascular injury are multifaceted. Basic studies have shown that intermittent hyperglycemia induces greater oxidative stress and apoptosis in endothelial cells than sustained hyperglycemia [20]. Although hyperglycemia itself damages vascular tissue, evidence regarding the direct effects of GV is limited [21]. GV has also been reported to enhance the formation of advanced glycation end-products, upregulate proinflammatory cytokines, and impair endothelial function [11,22]. In animal models, glucose fluctuations accelerate atherosclerotic plaque formation compared with sustained hyperglycemia of similar mean levels [23]. These findings support the notion that GV exerts harmful vascular effects beyond those attributable to chronic hyperglycemia. Clinically, a subanalysis of the Study to Prevent NIDDM trial suggested that postprandial hyperglycemia reduction with acarbose lowered cardiovascular events, but subsequent large-scale trials did not confirm this finding [24]. These discrepancies imply that GV may influence vascular events through indirect mechanisms, including oxidative stress, inflammation, and autonomic imbalance.

Autonomic dysregulation, particularly sympathetic overactivity, has been proposed as a key contributor to diabetic vascular disease [25,26]. Di Flaviani et al. showed that fine GV affects sympathovagal balance in patients with T2D [27]. In this context, GV may serve not only as a metabolic marker but also as a regulator of autonomic activity. SGLT2 inhibitors have been reported to reduce sympathetic nervous activation [28,29], which could partly explain their vascular protective properties. We have previously shown that SGLT2 inhibitors suppress urinary metanephrine, a metabolite of norepinephrine, suggesting reduced sympathetic tone [30]. Thus, luseogliflozin may indirectly attenuate excessive sympathetic nervous activity by reducing basal GV, thereby suppressing vascular complications.

This study has several limitations. The small sample size, single-center design, and short follow-up restrict generalizability. Patient characteristics such as residual insulin secretion, renal function, BMI, and concomitant medications were not fully assessed. Furthermore, sympathetic activity and vascular outcomes were not directly measured, and the observed mechanisms remain speculative. Since CGM was only performed during hospitalization, it is unclear whether the suppressive effect on nocturnal GV persists in the outpatient setting. Long-term prospective studies are needed to determine whether sustained suppression of basal GV with SGLT2 inhibitors contributes to vascular protection and a reduction in cardiovascular events.

## Conclusions

This study evaluated the effect of luseogliflozin, an SGLT2 inhibitor, on nocturnal basal glucose fluctuations in patients with type 2 diabetes using CGM. The CV significantly decreased after administration. Since glucose variability is linked to sympathetic activity and vascular damage, SGLT2 inhibitors may contribute to vascular protection. This is the first report showing suppression of basal nocturnal glucose fluctuations, warranting further investigation.
